# Cadmium and lead accumulation in important food crops due to wastewater irrigation: Pollution index and health risks assessment

**DOI:** 10.1016/j.heliyon.2024.e24712

**Published:** 2024-01-20

**Authors:** Yousef Alhaj Hamoud, Hiba Shaghaleh, Muhammad Zia-ur-Rehman, Muhammad Rizwan, Muhammad Umair, Muhammad Usman, Muhammad Ashar Ayub, Umair Riaz, Ghalia S.H. Alnusairi, Suliman Mohammed Suliman Alghanem

**Affiliations:** a The National Key Laboratory of Water Disaster Prevention and College of Hydrology and Water Resources, Hohai University, Nanjing, 210098, China; b Key Lab of Integrated Regulation and Resource Development on Shallow Lakes, Ministry of Education, College of Environment, Hohai University, Nanjing, 210098, China; c Institute of Soil & Environmental Sciences, University of Agriculture, Faisalabad, 38000, Pakistan; d Department of Environmental Sciences, Government College University, Faisalabad, 38000, Pakistan; e Institute of Agro-Industry and Environment, The Islamia University of Bahawalpur, 63100, Punjab, Pakistan; f Department of Biology, College of Science, Jouf University, Sakaka, 2014, Saudi Arabia; g Department of Biology, College of Science, Qassim University, Burydah, 52571, Saudi Arabia

**Keywords:** Bioavailability, Health risks, Target hazard quotient, Permissible limits, *Allium cepa*, *Medicago sativa*

## Abstract

The contamination of farm soils with heavy metals (HMs) has raised significant concerns due to the increased bioavailability and accumulation of HMs in agricultural food crops. To address this issue, a survey experiment was conducted in the suburbs of Multan and Faisalabad to investigate the spatial distribution, bioaccumulation, translocation, and health risks of cadmium (Cd) and lead (Pb) in agricultural crops. The results show a considerable concentration of Cd and Pb in soils irrigated with wastewater, even though these levels were below the permissible limits in water and soil matrices. The pollution index for Cd was mostly greater than 1 at the selected sites, indicating its accumulation in soil over time due to wastewater irrigation. Conversely, the pollution index for Pb was below 1 at all sites. Among the plants, *Zea mays* accumulated the highest concentration of Cd and Pb. The translocation factor from soil to root was highest for *Brassica olearecea* (7.037 for Cd) and *Zea mays* (6.383 for Pb). The target hazard quotient (THQ) value of Cd exceeded the non-carcinogenic limit for most vegetables. The highest value was found in *Allium cepa* (5.256) and the lowest in *Allium sativum* (0.040). In contrast, the THQ level of Pb was below the non-carcinogenic limit for most vegetables, except for *Allium cepa* (1.479), *Solanum lycopersicum* (1.367), and *Solanum tuberosum* (1.326). The study highlights that *Allium cepa* poses the highest health risk for humans, while *Medicago sativa* poses the highest risk for animals due to Cd and Pb contamination. These results underscore the urgent need for effective measures to mitigate the health risks associated with HM contamination in crops and soils.

## 1 Introduction

Toxic heavy metals, such as lead (Pb) and cadmium (Cd), are continuously accumulating in agricultural soils due to unmanaged anthropogenic activities [1,2], posing a threat to the safety of the native environment [3]. The global production of Cd reached up to 24,900 metric tons in 2015, as reported by the British Geological Survey [4]. The Cd naturally occurs in the earth's crust and sedimentary rocks, ranging from 0.1 to 15 mg kg^−1^ [5]. Similarly, Pb is abundantly present in the earth's crust, with concentrations of up to 14 mg kg^−1^ [6], and commonly found in ores containing silver (Ag), copper (Cu), and zinc (Zn) [7]. These metals originate from various sources, including mining, industrialization, and agricultural inputs [[8], [9], [10]]. However, untreated municipal wastewater has emerged as a significant contributor to heavy metal inputs in the environment [11,12], containing a substantial amount of pollutants from large industries, small dying units, houses, and hospitals [13,14]. Farmers often opt for wastewater irrigation due to its availability, nutrient enrichment, and cost-effectiveness [15]. The wastewater contains certain heavy metal ions like Zn, Cu, and manganese (Mn), which are essential for the metabolic activities of plants and animals at optimal concentrations. Other elements, such as Pb and Cd, are harmful even at lowest concentrations [16]. Plants primarily absorb heavy metals from the soil solution via roots, leading to their accumulation in plant tissues and posing potential health risks for consumers [5,17]. The urgency to comprehend the human exposure risk of toxic heavy metals in contaminated soil and implement timely risk management measures to prevent health hazards is due to the significant threats posed by heavy metal contamination to the environment and public health [18]. The widespread contamination of Cd and Pb in the environment, particularly in agricultural soils, indicates the urgent need to address the risks posed by heavy metal accumulation [19].

Human health-risk assessment models play a crucial role in determining the degree of harm posed by soil heavy metals to the human body [15]. These models aid in evaluating the health risks of heavy metals from various sources and identifying primary pollution sources, contributing to the development of targeted risk management strategies [16]. Additionally, the ecological and potential health risk assessment of heavy metals in soil and food crops is vital to understanding the risks to humans through the food chain and mitigating potential health hazards [20]. The emergence of environmental pollution-related public health hazards has prompted several countries to establish a health risk assessment (HRA) framework to quantify the health hazards. The HRA encompasses a comprehensive system of theories, methodologies, and tools used to estimate the nature and probability of adverse health effects in humans [21] exposed to toxic pollutants in the environment. Different agencies, like the National Research Council (NRC), proposed the definition and preliminary framework for HRA in 1983, leading to the gradual development of a four-step risk assessment model by the United States Environmental Protection Agency (USEPA) [22].

Extensive wastewater irrigation has been practiced in the suburbs of Multan and Faisalabad, Pakistan, leading to the contamination of soils with heavy metals [23,24]. However, a comprehensive study has not been conducted to evaluate the contamination status of soils irrigated with wastewater, particularly concerning human and animal health risk models, highlighting the health hazards to humans by consuming heavy metal-contaminated food. It was hypothesized that vegetables, cereals, and fodders grown on wastewater-irrigated soils may accumulate excess Cd and Pb, which may cause human and animal health risks. The main aims of the present survey study were: i) to assess the Cd and Pb contamination in wastewater and bioavailable fractions in the soil of different depths, ii) to find the bioaccumulation and translocation of Cd and Pb from soil to plant parts, iii) to assess the exposure and health risk to the population through various health risk assessment models.

## 2 Materials and methods

### 2.1 Survey area and sampling

The survey experiment was conducted in the suburbs of two industrial cities, Faisalabad (31.4504° N, 73.1350° E) and Multan (30.1575° N, 71.5249° E), where agricultural fields were irrigated with wastewater. The specific locations were chosen to collect water, soil, and plant samples during January and April 2019. Employing a simple random sampling method, collected samples were brought to the wirehouse at the Institute of Soil and Environmental Sciences (ISES), University of Agriculture, Faisalabad. The subsequent analysis of these samples was conducted at the Soil and Water Chemistry Laboratory (SWCL) within ISES. The detailed methodologies for the collection, preparation, and analysis of water, soil, and plant samples are as follows.

#### 2.1.1 Water samples collection and analysis

To collect underground and irrigation water samples, clean 1 L bottles were used. The samples were stored in the refrigerator to prevent redox changes caused by microbes. Two to three drops of 0.01 % hydrochloric acid were added to each sample to prevent the adsorption and precipitation of metal ions. Before analysis, the samples were filtered through the Whatman 42 filter paper. Heavy metals were determined in the water using an atomic absorption spectrometer (Analytik Jena novAA 800 F).

#### 2.1.2 Soil samples collection and analysis

Soil samples were collected from two depths (0–15 and 15–30 cm) in vegetable and crop fields. The sampling sites were selected uniformly, and a composite soil sample of 1 kg was collected by mixing sub-samples from each field. The collected soil samples were sealed in clean polythene bags and transported to the laboratory for further processing. In the laboratory, the soil samples were air-dried for 24 h to dry the soil samples. The samples were sieved using a 2.0 mm mesh size sieve. Each sieved sample weighed 10 g in a falcon tube, and 20 ml of ammonium bicarbonate diethylenetriaminepentaacetic acid (AB-DTPA) solution was added to the falcon tube. The suspension was uniformly shaken using a mechanical shaker for 5 min and filtered through the Whatman 42 filter paper [25]. The resulting filtrates were stored in clean plastic bottles for subsequent analysis. Heavy metals were analyzed in the soil using an atomic absorption spectrophotometer (Analytik Jena novAA 800 F).

#### 2.1.3 Plant sample collection and analysis

Plant samples, including various crops, vegetables, and trees, were collected based on availability. To prepare the samples for analysis, all plant samples were brought to the SWCL and washed with distilled water to remove dust and soil particles. A sharp knife was used to cut the plant samples into root, shoot, and edible portions. The washed plant parts were air-dried in the lab. The air-dried samples were oven-dried at 60 °C until a constant dry weight was achieved. The plant samples were ground to make leaves, roots, and woody stems powder. For each crop or vegetable, precisely 1 g of sample was placed in digestion flasks. The samples were digested in a solution of perchloric acid and nitric acid (in a ratio of 1:3) at a temperature range of 200–300 °C. The samples were cooled at room temperature and filtered through the Whatman 42 filter paper. The filtrate was analyzed using an atomic absorption spectrophotometer (Analytik Jena novAA 800 F) to detect Cd and Pb in plant parts [26].

### 2.2 Accumulation of heavy metals in soil and plant

The bio-accumulation factor (BAF) of Cd and Pb was calculated by the following equation (Eq. 1), as the proportion of Cd and Pb concentration in the part of the plant to the corresponding plant-cultivated soil.

(1) BAF = metal in plant tissues/metal in soil

The Cd and Pb translocation factor (TF) for different plant tissues was calculated by equations (Eq. 2 and 3), reported by Rezvani et al. [27],.

(2) TF (root to shoot) = metal in shoot/metal in root

(3) TF (soil to root) = metal in root/metal in soil

The soil's pollution index (PI) was calculated using the ratio of heavy metals (Cd and Pb) concentration in the sample to the background value of heavy metals in the soil. The formula for the PI is as follows (Eq. (4)),:

(4) PI = metal in sample/background value of metal ion

The background value for Cd was 0.3 mg kg^−1^, and Pb's was 20 mg kg^−1^ [28].

### 2.3 Exposure assessment

The USEPA health risk assessment models have been extensively used to determine carcinogenic and non-carcinogenic risks of Cd and Pb in humans [28] and animals [31] through oral ingestion.

#### 2.3.1 Daily intake of metals for humans and cattle

The daily intake of heavy metals (DIM) for humans and cattle was determined by the equation (Eq. 5):

(5) DIM (mg kg^−1^ day^−1^) = C_m_ × C(factor) × D (daily vegetable or fodder intake)/BW (average body weight)

The metal concentration in vegetables (mg kg^−1^) on a dry weight basis is represented as C_m_. “C” is the conversion factor of fresh weight of vegetables to dry weight, which was 0.085, as mentioned by Rattan et al. [11]. In the equation, BW represents body weight, considered 62 kg, based on Walpole et al. [32]. The daily vegetable intake per capita is detailed in Table S1, which was extracted from data reported by Antoine et al. [33]. For cattle, the daily intake of metals was calculated by the above equation, where C (factor) was the same as used above, while C_m_ was fodder metal concentration Schubert et al. [34] and Saha et al. [35]. Unlike humans, an 80 kg day^−1^ cumulative fodder intake value of D was taken for cattle.

### 2.4 Risk characterization

#### 2.4.1 Health quotient for humans and cattle

Health quotient (HQ) for both humans and cattle was estimated by following the equation (Eq. 6) as reported by Iqbal et al. [30]:

(6) HQ = Daily Intake of Metal (DIM)/Oral Reference Dose (R_f_D)

The RfD (reference dose) values used for Cd and Pb were 0.001 and 0.004 mg kg^−1^ day^−1^, respectively, for humans. The oral reference doses for Cd and Pb for cattle were calculated as 0.01 and 0.04 mg kg^−1^ day^−1^, respectively, using specific formulas and factors.

The cumulative health risk from all vegetables was assessed by summing the individual HQ values for both metals [36]. A cumulative HQ value exceeding 1 indicates a potential health risk (as in Eq. (7)).

(7) $$\text{Cumulative}\;\text{HQ}={\text{HQ}}_{\text{Veg}\;1\;\left(\text{Cd}+\text{Pb}\right)}+{\text{HQ}}_{\text{Veg}\;2\;\left(\text{Cd}+\text{Pb}\right)+\ldots \ldots .+}{\text{HQ}}_{\text{Veg}\;n\;\left(\text{Cd}+\text{Pb}\right)}$$

### 2.5 Target hazard quotient for humans

In this study, THQ values for the population consuming vegetables from irrigated perimeters were calculated using the equation as reported by Antoine et al. [33] based on the USEPA methodology as given in the equation (Eq. 8).

(8) THQ =(E_f_ × C_m_ × E_d_ × F_i_)/(R_f_D × AE_t_ × BW)

Where E_f_: Exposure frequency (taken as 365 days), C_m_: Heavy metals concentration in vegetables (mg kg^−1^ DW), E_d_: Exposure duration (taken as 70 years), F_i_: Per day consumption of respective vegetable, R_f_D: Oral reference dose, AE_t_: Average exposure time (365 days × 70 years), BW: body weight (62 kg).

### 2.6 Total target hazard quotient for humans

It has been reported that the additive effect of exposure to multiple toxic metals can have more health hazards [36], So there is also a need to evaluate the additive risks of Cd and Pb. The total target hazard quotient (TTHQ) of Cd and Pb for each vegetable was calculated by the sum of THQ for individual metals (Eq. (9)).

(9) $${\text{TTHQ}}_{\text{Veg}\;1}={\;\text{THQ}}_{\text{Cd}}+{\text{THQ}}_{\text{Pb}}$$

### 2.7 Health hazard index for humans

The health hazard index (HHI) assessed the cumulative non-carcinogenic health risks of consuming contaminated vegetables containing multiple heavy metals. The HHI is calculated by Saha et al. [35]. The equation (Eq. 10) used for HHI is as follows:

(10) $$\text{HHI}={\text{TTHQ}}_{\text{veg}\;1}+{\;\text{TTHQ}}_{\text{veg}\;2}+{\text{TTHQ}}_{\text{veg}\;3}+\cdots \cdots {+\text{TTHQ}}_{\text{veg}\;n}$$

Suppose the HHI value is greater than 1. In that case, it indicates that the cumulative non-carcinogenic health risks associated with consuming contaminated vegetables are unacceptable and potentially threaten human health [9].

### 2.8 Statistical analysis

Statistical analysis was conducted using Minitab 17 Statistical Software to analyze the collected data [37]]. In addition to Minitab, means and standard errors were calculated in Excel 2016.

## 3 Results and discussion

### 3.1 Spatial distribution of Cd and Pd in soil and irrigational water

The contamination of Cd and Pb in the agricultural fields was assessed, and results are summarized in Table 1 and Table S2. Results show the spatial variation of Cd and Pb in soil and water samples in two main industrial cities of Punjab, Pakistan. Overall, it was observed that the concentration of Cd (AB-DTPA extractable) in the soil at a depth of 0–15 cm and 15–30 cm ranged from 0.19 to 1.09 mg kg^−1^ and 0.09–0.64 mg kg^−1^, respectively, in suburban of Multan. The Pb concentration at the same depths ranged from 0.27 to 2.19 mg kg^−1^ and 0.14–1.46 mg kg^−1^, respectively. Similarly, in Faisalabad, the soil concentrations of Cd at a depth of 0–15 cm and 15–30 cm were found to range from 1.22 to 1.69 mg kg^−1^ and 0.45–0.65 mg kg^−1^, respectively, while the concentration of Pb was ranged from 2.87 to 4.01 mg kg^−1^ and 1.14–1.51 mg kg^−1^ at both depths. It was observed that the bio-available concentration of both Cd and Pb in soil was below the permissible limits, as given in Table S2. Furthermore, the upper soil layer (0–15 cm) had higher concentrations of Cd and Pb than the lower depths (15–30 cm), as shown in Table 1. Similarly, the analysis of raw effluents used for irrigation showed that all the samples contained Pb and Cd, but these concentrations were also below the allowable limits for irrigation. The contamination of Cd and Pb was quite variable depending upon the sources and dilution mixings. In Multan, wastewater was found to have Cd and Pb concentrations of 0.007–0.01 and 0.14–0.78 mg L^−1^, respectively. In Faisalabad, sewage water was found to have Cd and Pb concentrations in the range of 0.01–0.011 and 0.5–0.62 mg L^−1^, respectively. This trend has been reported in different studies, reflecting the low mobility of Cd and Pb with leaching water [13,38]. The concentration of Cd and Pb in soil can increase due to wastewater irrigation. Studies have shown that heavy metals such as Cd and Pb can accumulate in soil and crops due to using contaminated water for irrigation [[39], [40], [41], [42]]. The concentration of Cd and Pb in soil can also be affected by weathering and chemical reactions in the soil [7]. Overall, the presence of Cd and Pb in wastewater and their subsequent impact on soil and crop quality underscores the importance of monitoring and managing heavy metal contamination in water sources used for irrigation to mitigate potential health and environmental risks.

**Table 1:** Spatial concentration of Cd and Pb in soil and water samples collected from suburban Multan and Faisalabad.

Site No.	Area	Coordinates	*Wastewater Concentration (mg L*^*−*^*^1^)*		*AB-DTPA extractable concentration (mg kg*^*−*^*^1^)*			
					0–15 cm		15–30 cm	
			Cd	Pb	Cd	Pb	Cd	Pb
*1.*	Multan	30 11 47 N 71 32 47 E	0.009	0.31	0.22	0.51	0.09	0.28
*2.*		30 11 11 N 71 31 04 E	0.01	0.50	0.84	1.33	0.10	0.64
*3.*		30 10 01 N 71 30 24 E	0.009	0.44	0.44	0.54	0.11	0.16
*4.*		30 09 18 N 71 30 10 E	0.008	0.52	NA	NA	NA	NA
*5.*		30 07 21 N 71 21 41 E	0.008	0.78	0.19	0.45	0.12	0.14
*6.*		30 07 20 N 71 26 20 E	0.007	0.77	0.20	0.86	0.10	0.71
*7.*		30 06 44 N 71 26 20 E	0.007	0.14	0.27	0.27	0.19	0.19
*8.*		30 09 16 N 71 28 49 E	0.01	0.71	1.09	1.40	0.64	1.14
*9.*		30 09 00 N 71 28 02 E	0.01	0.43	0.94	2.19	0.44	1.46
*10.*		30 08 55 N 71 27 39 E	0.01	0.20	1.09	1.01	0.28	0.35
*11.*		30 08 46 N 71 27 00 E	0.01	0.46	0.66	1.46	0.54	0.91
*12.*		30 14 46 N 71 25 21 E	0.01	0.71	0.86	1.48	0.40	0.99
*13.*		30 14 08 N 71 25 38 E	0.01	0.71	0.82	0.99	0.32	0.60
*14.*	Faisalabad	31 27 45 N 72 58 35 E	0.011	0.50	1.22	3.88	0.53	1.51
*15.*		31 28 05 N 72 56 07 E	0.011	0.50	1.24	3.51	0.45	1.14
*16.*		31 30 15 N 73 07 16 E	0.01	0.62	1.69	4.01	0.62	1.51
*17.*		31 18 24 N 73 07 06 E	0.01	0.55	1.35	2.87	0.65	1.43
***Minimum***	0.007	0.14	0.19	0.27	0.09	0.14		
***Maximum***	0.011	0.78	1.69	4.01	0.65	1.51		
***Range***	0.007–0.011	0.14–0.78	0.19–1.69	0.27–4.01	0.09–0.65	0.14–1.51		

Mean Cd and Pb concentrations in wastewater and soils collected from different wastewater-irrigated agriculture fields at selected sites. NA: no soil sample available.

### 3.2 Soil pollution index for Cd and Pb at selected sites

**Fig. 1:**
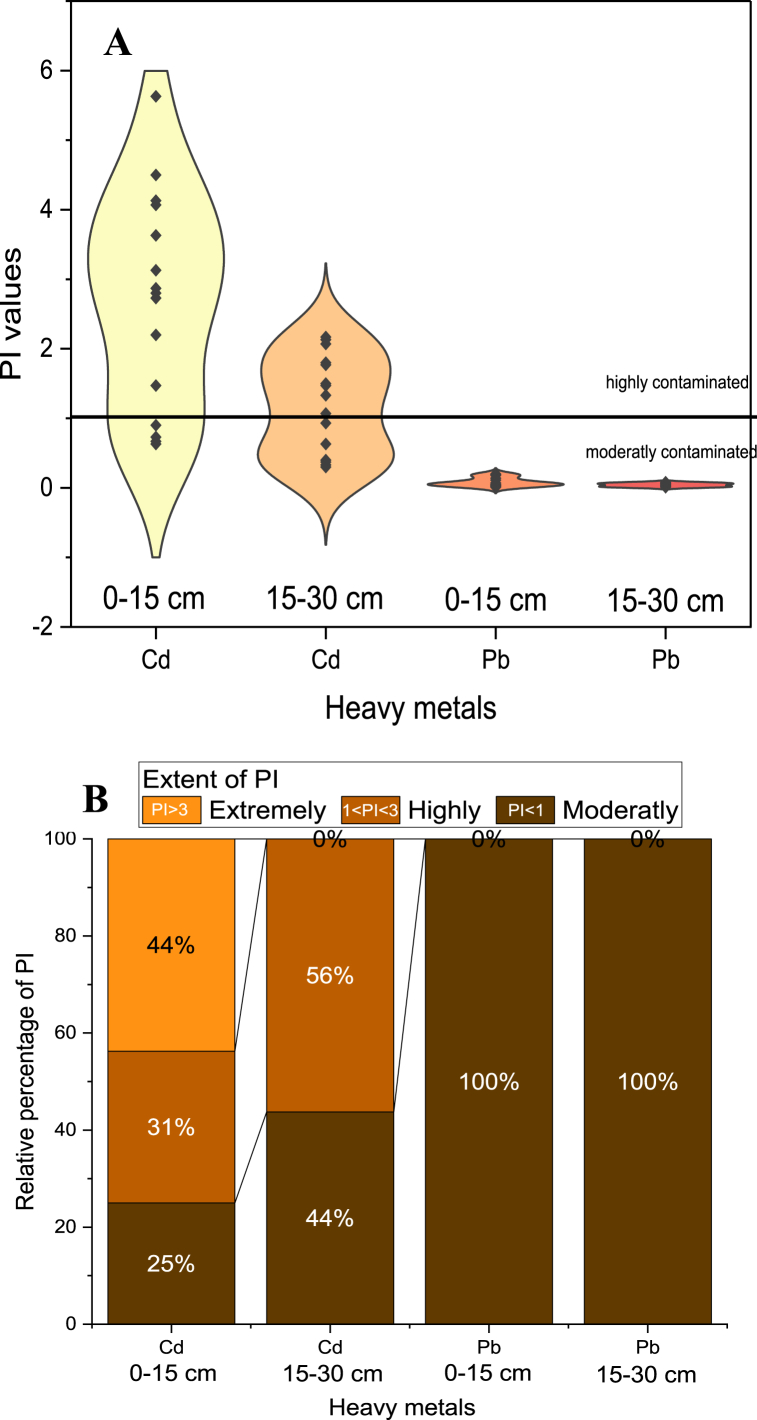
(A) Pollution index values of Cd and Pb at selected wastewater irrigated sites, (B) Relative percentage of pollution index concerning level of contamination at wastewater irrigated sites.

The PI for Cd and Pb in the soil at selected sites was assessed to understand the extent of heavy metal contamination and its potential ecological and human health risks (Fig. 1A and B). Chemical speciation and bioavailability of Cd and Pb in soil showed significant changes in Cd and Pb fractions, emphasizing the need to assess the PI to understand the bioavailability and potential risks associated with Cd and Pb in soil. The PI for Cd was found to range from 0.63 to 5.63 and 0.30 to 2.17 for the 0–15 cm and 15–30 cm soil depths, respectively. The range of PI value for Pb was found to be 0.01 to 0.20 and 0.01 to 0.08 for the 0–15 cm and 15–30 cm soil depths, respectively. The PI value greater than 1 for Cd indicated its accumulation at wastewater-irrigated sites compared to its background value of 0.3 mg kg^−1^. Conversely, a PI value less than 1 for Pb indicated a decrease in Pb over time (Fig. 1A). The data suggests higher Cd pollution in the suburbs of Faisalabad compared to Multan, while Pb has decreased over time at all sites compared to its background value of 20 mg kg^−1^. Cd's PI value ranged from 0.63 to 5.63 in the root zone, which is a serious concern regarding Cd accumulation in soil over time due to wastewater irrigation. Meanwhile, the PI of Pb was found to be highest up to 0.20 in the root zone, which was in moderate contamination. These findings are consistent with previous studies that have reported the impact of heavy metal concentrations on agricultural yields due to uptake from irrigation water, as well as the ecological and health implications of elevated levels of Cd and Pb in soil and wastewater [[43], [44], [45], [46], [47]]. Assessing the PI for Cd and Pb in soil at selected sites is crucial for understanding the extent of heavy metal contamination, evaluating potential ecological and human health risks, and informing appropriate remediation and management strategies.

### 3.3 Concentration of Cd and Pb in crop tissues

**Fig. 2:**
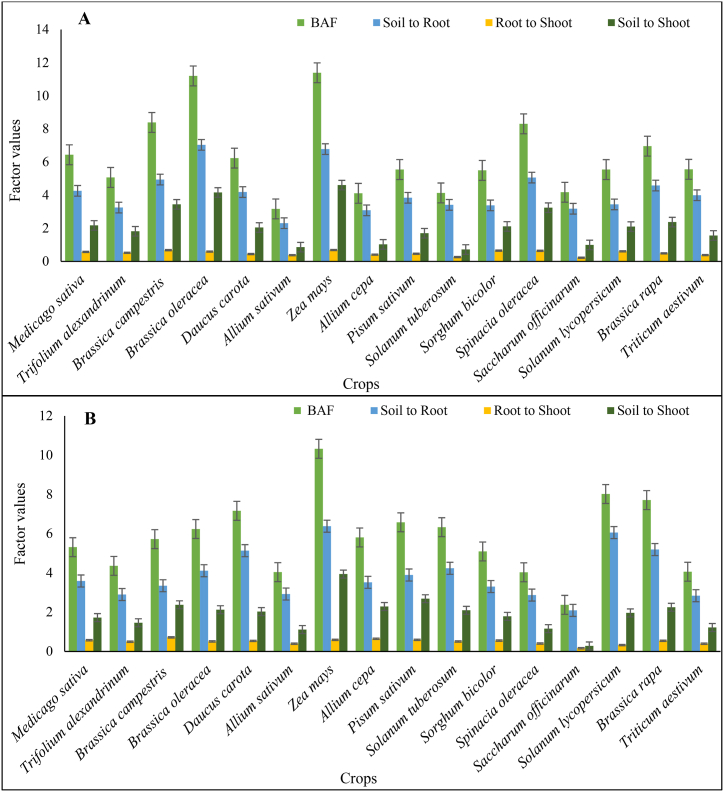
Translocation factor values of Cd (A) and Pb (B) in different vegetable and fodder crops at Multan. Bars indicate the mean values of the bioaccumulation factor and translocation factor (soil to roots, roots to shoot, and soil shoot) in different plants, and error bars show the standard deviation among the number of samples analyzed.

The concentration of Cd and Pb in crop tissues as cultivated in wastewater irrigation fields highlighted the impact of Cd and Pb concentrations on agricultural yields and the transfer of these heavy metals from soil to the edible parts of crops [3,4,7]. The current experiment observed that most of the crops were at their booting stage (Table 2). The overall range of Cd concentration in roots remained between 2.35 and 4.03 mg kg^−1^, with the maximum Cd found in *Medicago sativa* roots and the minimum in *Solanum tuberosum*. The range of Pb concentration in roots was 2.64–14.6 mg kg^−1^; the maximum Pb was found in *Solanum lycopersicum* and the minimum in *Spinacia oleracea*. In the shoot samples, the overall range of Cd concentration remained 0.59–2.47 mg kg^−1^; the maximum Cd was found in *Sorghum bicolor* shoot and the minimum in *Solanum Tuberosum*. The range of Pb concentration in shoots was 0.89–5.67 mg kg^−1^, with the maximum Pb in *Medicago sativa* shoot and the minimum in *Saccharum officinarum*. The data from Fig. 2A and B indicate that roots accumulated higher concentrations of Cd and Pb than shoots, possibly due to the direct interaction of roots with the soil solution. This observation is consistent with the findings of Jeelani et al. [48], who reported that plants accumulate heavy metals, such as Cd and Pb, more in their root tissues than in their shoot tissues. This is attributed to roots primarily coming into contact with the soil solution [46], which contains higher quantities of trace metals. Due to their restricted mobility within plants, these toxic metals, such as Pb and Cd, tend to reside in the roots rather than moving to the above-ground parts [49]. The findings of the current experiment collectively underscore the importance of monitoring and managing Cd and Pb concentrations in crop tissues, especially in wastewater irrigation, to ensure food safety and mitigate potential health and environmental risks.

**Table 2:** Spatial distribution of Cd and Pb in plant tissues under wastewater irrigation at selected sites of Multan and Faisalabad.

Crops	No. Samples	Underground parts (mg kg^−1^)				Aerial parts (mg kg^−1^)			
		Cd		Pb		Cd		Pb	
		Mean ± S.E	Range	Mean ± S.E	Range	Mean ± S.E	Range	Mean ± S.E	Range
*Brassica oleracea*	13	3.26 ± 0.12	2.70–4.07	3.83 ± 0.17	2.78–5.20	1.86 ± 0.13	1.15–2.49	1.92 ± 0.18	1.28–3.06
Brassica campestris	12	3.13 ± 0.08	2.77–3.65	2.73 ± 0.24	1.77–3.82	2.13 ± 0.07	1.75–2.58	1.97 ± 0.13	1.44–2.47
Brassica rapa	6	2.53 ± 0.05	2.38–2.64	3.63 ± 0.12	3.06–3.80	0.9 ± 0.14	0.73–1.62	1.6 ± 0.20	1.28–2.57
Allium cepa	6	3.48 ± 0.13	2.88–3.79	3.79 ± 0.03	3.68–3.89	0.63 ± 0.29	0.32–2.09	2.46 ± 0.04	2.37–2.60
Solanum lycopersicum	5	3.65 ± 0.19	3.18–4.04	14.6 ± 0.22	14.03–15.34	2.23 ± 0.02	2.18–2.28	4.73 ± 0.15	4.23–5.12
Solanum tuberosum	15	2.35 ± 0.12	2.01–3.00	5.61 ± 0.07	5.23–5.95	0.59 ± 0.08	0.28–1.07	2.24 ± 0.13	1.63–2.99
Spinacia oleracea	3	3.34 ± 0.09	3.18–3.48	2.64 ± 0.13	2.43–2.88	2.14 ± 0.04	2.09–2.23	1.07 ± 0.03	1.02–1.13
Daucus carota	22	2.58 ± 0.08	1.95–3.31	4.56 ± 0.17	2.84–5.95	0.95 ± 0.10	0.41–1.69	1.85 ± 0.07	1.38–2.44
Allium sativum	8	2.48 ± 0.03	2.34–2.60	4.65 ± 0.03	4.58–4.84	0.89 ± 0.06	0.70–1.17	1.75 ± 0.14	1.37–2.23
Pisum sativum	17	2.61 ± 0.16	1.63–3.90	4.56 ± 0.14	3.65–5.25	1.1 ± 0.15	0.25–2.17	2.92 ± 0.23	1.95–4.39
*Triticum aestivum*	38	2.54 ± 0.12	1.37–4.00	4.42 ± 0.27	2.06–8.00	0.63 ± 0.10	0.20–2.00	1.37 ± 0.15	0.35–2.96
Medicago sativa	20	4.03 ± 0.19	3.10–5.36	10.47 ± 0.66	5.12–14.00	2.16 ± 0.15	1.07–2.93	5.67 ± 0.55	1.99–10.74
Saccharum officinarum	18	2.88 ± 0.12	2.10–3.40	7.81 ± 0.79	3.46–14.47	0.63 ± 0.11	0.19–1.34	0.89 ± 0.27	0.21–3.71
Zea mays	12	3.37 ± 0.05	3.19–3.76	7.46 ± 0.35	5.74–9.85	2.26 ± 0.07	2.02–2.78	4.6 ± 0.13	3.78–5.20
Sorghum bicolor	29	3.66 ± 0.19	2.12–5.23	7.7 ± 0.77	2.51–13.70	2.47 ± 0.17	1.01–3.80	4.51 ± 0.51	1.18–9.12
Trifolium alexandrinum	14	2.44 ± 0.17	1.51–3.51	4.36 ± 0.20	2.52–5.16	1.06 ± 0.14	0.43–2.23	1.88 ± 0.11	1.07–2.41
***Minimum***		**2.35 ± 0.12**	**1.37**	**2.64 ± 0.13**	**1.77**	**0.59**	**0.19**	**0.89**	**0.21**
***Maximum***		**4.03 ± 0.19**	**5.36**	**14.6 ± 0.22**	**15.34**	**2.47**	**2.18**	**5.67**	**4.23**
***Range***		**2.35–4.03**	**1.37–5.36**	**2.64–14.6**	**1.77–15.34**	**0.59–2.47**	**0.19–2.18**	**0.89–5.67**	**0.21–4.23**

### 3.4 Bioaccumulation and translocation of Cd and Pb in the field crops

**Fig. 3:**
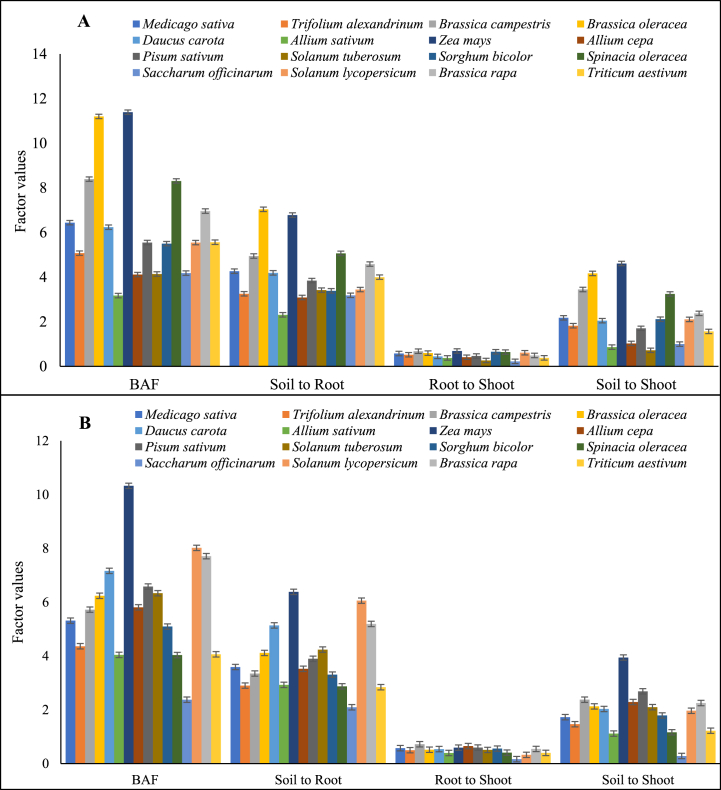
Bioaccumulation and translocation of Pb (A) and Cd (B) from soil to root, root to shoot, and soil to shoot in different crops at Faisalabad. Bars indicate the mean values of the bioaccumulation factor and translocation factor (soil to roots, roots to shoot, and soil shoot) in different plants, and error bars show the standard deviation among the number of samples analyzed.

Plants can accumulate higher quantities of heavy metals without exhibiting visible symptoms of toxicity [50,51]. The maximum translocation factor of soil to root was found for *Brassica oleracea* (7.037) and *Zea mays* (6.383) for Cd and Pb, respectively (Fig. 3A and B). Similarly, the translocation factor value for soil to shoot was greater than 1 for all crops and showed accumulation of Cd and Pb in aerial parts. However, the translocation factor for root to shoot was less than 1, showing that roots are major residing components for Cd and Pb and restrict their entry to aerial parts. The maximum translocation factor of root to shoot was found for *Zea mays* (0.689) and *Brassica campestris* (0.719) for Cd and Pb, respectively. Different plant species have a large variation regarding bioaccumulation and translocation factors. The translocation factor (Fig. 3A and B) for soil to root found is greater than 1, showing that roots have accumulated Cd and Pb concentrations to a high level. Numerous studies revealed that plant species potentially absorb and accumulate metals in their root and shoot tissues [49,52]. Genetic differences and labile metal contents in soils also affect metal accumulations in crop grain and shoot tissues [50,53]. The crops' ability to absorb metals depends on their ability to absorb metals and soil-solution-plant transfer factors [11]. In this study, highest bioaccumulation factor of Cd found for *Zea mays* followed by *Brassica oleracea* > *Brassica campestris* > *Spinacia oleracea* > *Brassica rapa* > *Medicago sativa* > *Daucus carota* > *Triticum aestivum* > *Pisum sativum* > *Solanum lycopersicum* > *Sorghum bicolor* > *Trifolium alexandrinum* > *Saccharum officinarum* > *Solanum Tuberosum* > *Allium cepa* > *Allium sativum*. Similarly, the highest bioaccumulation factor of Pb also found for *Zea mays* followed by *Solanum lycopersicum > Brassica rapa > Daucus carota > Pisum sativum > Solanum Tuberosum > Brassica oleracea > Allium cepa > Brassica campestris > Medicago sativa > Sorghum bicolor > Trifolium alexandrinum > Triticum aestivum > Allium sativum > Spinacia oleracea > Saccharum officinarum.*

**Fig. 4:**
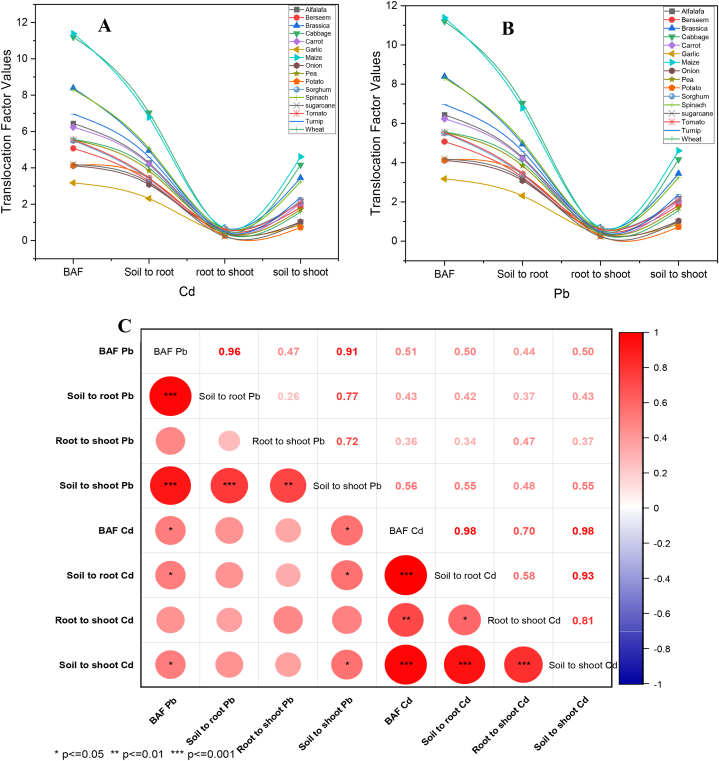
Cd (a) and Pb (b) translocation model in different crops and person correlation analysis of Cd and Pb bioaccumulation, translocation factors (soil to root, root to shoot, and soil to shoot) in different crops (c). * Showing the p-value less than 0.05, ** showing the p-value is less than 0.01, and *** showing the p-value less than 0.001.

The maximum BAF and TF were observed for *Zea mays* (Fig. 4A and B). This is consistent with previous studies that have reported the high potential of *Zea mays* for TF and BAF of heavy metals in its tissues [54,55]. The BAF value of Cd and Pb in crops growing in less contaminated soil was lesser than those growing in more contaminated soil only due to the lower Cd and Pb contamination in the soil of the earlier crop. The Cd and Pb uptake in crops depends on various factors, such as their bioavailability in soil solution, which depends on organic matter contents, soil pH, and metal speciation [55]. The current experiment had a positive correlation among TF and BAF of Cd and Pb (Fig. 4C). It was observed that Cd and Pb bioaccumulation in different vegetable and fodder crops is strongly correlated with translocation from soil to root, irrespective of the fact that roots were major residing components [56]. The translocation of Cd and Pb to aerial parts was found to be strongly dependent on the soil-to-root translocation factor [[57], [58], [59]]. The results highlighted the transfer characteristics of Cd and Pb from soil to the edible parts of various vegetable and field crop species, emphasizing the importance of controlling Cd and Pb concentrations in plants, particularly in the edible parts of crops, to ensure food safety.

### 3.5 Daily intake of metals by humans and cattle and health quotient

**Fig. 5:**
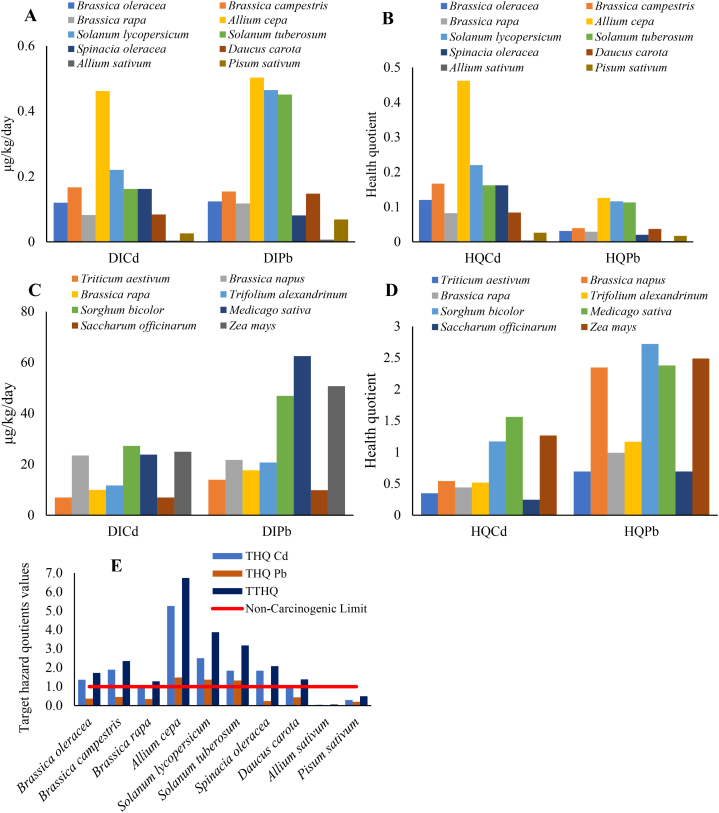
Daily intake of Cd and Pb (A) and health quotient (B) of humans associated with consumption of contaminated vegetables. Daily intake of metals (C) and cumulative Cd and Pb health quotient (D) in cattle from consumption of contaminated fodders crops. Target hazard quotients for humans from consuming contaminated vegetables (E). In figures, bars indicate the mean values.

The assessment of human and cattle health risks associated with consuming heavy metals such as Cd and Pb is a critical area of this study. Furthermore, this experiment explored the human health risk indices such as hazard quotient (HQ) and DIM, offering insights into the potential health risks associated with Cd and Pb exposure through food consumption. The DIM by humans is influenced by the concentration of metals in the edible portions of specific vegetables and their daily consumption values (Fig. 5A). For instance, the maximum DIM value of Cd was found for *Allium cepa* (0.4616 μg kg^−1^ day^−1^), followed by *Solanum lycopersicum*, *Brassica campestris*, *Solanum Tuberosum*, *Spinacia oleracea*, *Brassica oleracea*, *Daucus carota*, *Brassica rapa*, *Pisum sativum*, and *Allium sativum*. Similarly, the maximum DIM value of Pb was also found for *Allium cepa* (0.5027 μg kg^−1^ day^−1^), followed by *Solanum lycopersicum*, *Solanum Tuberosum*, *Brassica campestris*, *Daucus carota*, *Brassica oleracea*, *Brassica rapa*, *Spinacia oleracea*, *Pisum sativum*, and *Allium sativum*. These findings demonstrate the exposure assessment of Cd and Pb through the daily consumption of different vegetables. While some vegetables may have high concentrations of Cd and Pb, their low ingestion rates result in lower associated risks. Conversely, other vegetables may have relatively lower metal concentrations, but their higher DIM values pose greater risks due to their higher ingestion rates. Thus, consumption and concentration levels influence humans' DIM values. Furthermore, the highest HQ for humans was found for *Allium cepa* (0.587), followed by *Solanum lycopersicum*, *Solanum Tuberosum*, *Brassica campestris*, *Spinacia oleracea*, *Brassica oleracea*, *Daucus carota*, *Brassica rapa*, *Pisum sativum*, and *Allium sativum*. The HQ values for Cd and Pb in individual vegetables were less than 1 (Fig. 5B). However, the cumulative effect of these metals was calculated to be 2.016, indicating a potential risk to consumer health as its value is greater than 1. This model has been utilized in numerous studies to predict human health risks. It is important to note that the health quotient model may not fully represent the risk of consuming contaminated vegetables, as it does not account for long-term exposure. In our study, the human intake of Cd was reported to be highest through the consumption of *Allium cepa*. Besante et al. [60] also reported that Cd contamination of food has severe toxic effects on human health, especially in developing countries [51]. High concentrations of these metals in edible portions affect food quality and pose health risks, particularly to local consumers.

The DIM by cattle revealed that the maximum DIM value of Cd was found for *Sorghum bicolor* (0.0272 mg kg^−1^ day^−1^), followed by *Zea mays*, *Medicago sativa*, *Brassica napus*, *Trifolium alexandrinum*, *Brassica rapa*, *Saccharum officinarum*, and *Triticum aestivum*. Similarly, the maximum DIM value of Pb was found for *Medicago sativa* (0.062 mg kg^−1^ day^−1^), followed by *Zea mays*, *Sorghum bicolor*, *Brassica napus*, *Trifolium alexandrinum*, *Brassica rapa*, *Triticum aestivum*, and *Saccharum officinarum*. Among these fodders, *Sorghum bicolor*, *Zea mays*, and *Medicago sativa* were identified as the riskiest fodder for animals. Notably, cattle have a high fodder intake rate, resulting in significantly higher Cd and Pb DIM values than humans. The HQ values for cattle were also calculated using the same model as humans, as there is no age limit for cattle, making this model the best predictor of their health risk. The HQ values of Cd were found to be higher than those for Pb (Fig. 5C). Although the fodder consumption rate was not specified for individual fodder, the average ingestion rate of 80 kg was used for all types of fodder. The highest HQ was found for *Sorghum bicolor* (2.722) and *Medicago sativa* (1.562) for Cd and Pb, respectively. However, the total HQ was found to be highest for *Medicago sativa* (3.943), followed by *Sorghum bicolor*, *Zea mays*, *Brassica napus*, *Trifolium alexandrinum*, *Brassica rapa*, *Triticum aestivum*, and *Saccharum officinarum*. The experiment revealed that cattle are at significant risk due to consuming Cd and Pb-contaminated fodder (Fig. 5D). Iqbal [31] also reported that heavy metal contamination of maize crops irrigated with wastewater led to health risks for cattle, making them weak and more vulnerable to diseases [29]. Estimating potentially toxic heavy metal accumulation in cattle tissues is crucial for livestock management and human intake of meat [61].

### 3.6 Target hazard quotient and total target hazard quotient for humans

The THQ evaluates the potential health risks associated with exposure to toxic elements. The THQ values greater than 1 indicate a potential health risk, while values less than or equal to 1 suggest that adverse effects are not likely to occur, and the hazard can be considered negligible [33]. It provides valuable insights into the potential health hazards associated with exposure to toxic elements. It helps determine the likelihood of adverse health effects occurring due to exposure [43]. In the current experiment, it was observed that THQ for Cd exceeded the non-carcinogenic limit for most vegetables, with the highest value found in *Allium cepa* (5.256) and the lowest in *Allium sativum* (0.040) (Fig. 5E). The variation in THQ levels among these vegetables was attributed to differences in vegetable consumption rates and Cd concentrations. In contrast, the THQ level of Pb was below the non-carcinogenic limit for most vegetables, except for *Allium cepa* (1.479), *Solanum lycopersicum* (1.367), and *Solanum Tuberosum* (1.326). The difference in THQ values between Cd and Pb is due to a wide range of differences in oral reference dose, with Cd being four times lower than that of Pb, resulting in a higher THQ for Cd than for Pb. The TTHQ value was highest for *Allium cepa* (6.735), followed by *Solanum lycopersicum* (3.872), *Solanum Tuberosum* (3.171), *Brassica campestris* (2.351), *Spinacia oleracea* (2.081), *Brassica oleracea* (1.727), *Daucus carota* (1.385), *Brassica rapa* (1.278), *Pisum sativum* (0.494), and *Allium sativum* (0.059). The THQ and TTHQ values > 1 indicate a significant non-carcinogenic health risk from consuming wastewater-irrigated vegetables.

### 3.7 Health hazard index of Cd and Pb for humans

The HHI from consuming vegetables contaminated with Cd and Pb over an average lifespan of 70 years showed a cumulative HHI value of 23.152, significantly exceeding the tolerable limit 1. This indicates an unavoidable non-carcinogenic health risk from these vegetables over an individual's lifetime consumption. Therefore, a substantial health risk is associated with the wastewater irrigation of vegetables and their human consumption [2,3,6,61,62]. The exposure of humans to Cd and Pb through ingestion, dermal adsorption, and inhalation has been reported to be higher in developing countries, leading to detrimental effects on human health. The ingress of Cd and Pb through contaminated food and water is the main source of exposure, resulting in the bioaccumulation of these metals in vegetable and fodder crops, ultimately leading to their accumulation in edible parts [35,44,53,62]. The disturbance of enzyme activity, especially thiol group-containing enzymes and antioxidants, has been observed due to heavy metal ions like Cd, Pb, As, Cr, and Hg [63]. The findings from various studies have highlighted the potential health risks associated with the long-term ingestion of vegetables contaminated with heavy metals, emphasizing the need for continuous monitoring and effective measures to mitigate these health risks [2,3,20,58,60].

## 4 Conclusions

The current survey experiment found that the concentration of cadmium and lead in vegetables and fodder crops irrigated with wastewater exceeded permissible limits. The wastewater used for irrigation and their related soils also had significant cadmium and lead concentrations. These metals enter the food crops directly from contaminated soil, wastewater, and/or polluted air, and the soil-to-plant translocation of cadmium and lead is a crucial step in their ingress into the food chain. Contamination of vegetables and fodder crops poses health risks to humans and cattle. The study recommends regular monitoring of heavy metal contamination in soil, water, and plant samples and conducting surveys to measure the per capita daily intake of vegetables and other food items by the local population. Proper wastewater treatment before discharge into freshwater streams or agricultural soils is also recommended. Additionally, the study suggests measuring the level of contamination in all environmental matrices regularly to assess health hazards and suggest control measures. Such environmental measurements provide an actual scenario of metal distribution, principal sources, their fate in the environment, and bioaccumulation in the food chain.

## Funding

This research work was financially supported by the Jiangsu Funding Program for Excellent Postdoctoral Talent (2023ZB897 and 2023ZB869), the 10.13039/501100007654University of Agriculture Faisalabad, and the Foreign Youth Talent Project (2019/423402).

## CRediT authorship contribution statement

**Yousef Alhaj Hamoud:** Writing – review & editing, Writing – original draft, Validation, Methodology, Conceptualization. **Hiba Shaghaleh:** Writing – review & editing, Writing – original draft, Methodology, Conceptualization. **Muhammad Zia-ur-Rehman:** Writing – review & editing, Writing – original draft, Supervision, Project administration, Methodology, Conceptualization. **Muhammad Rizwan:** Writing – review & editing, Project administration, Conceptualization. **Muhammad Umair:** Formal analysis, Data curation. **Muhammad Usman:** Formal analysis, Data curation. **Muhammad Ashar Ayub:** Formal analysis, Data curation. **Umair Riaz:** Formal analysis, Data curation. **Ghalia S.H. Alnusairi:** Writing – review & editing. **Suliman Suliman M.S. Alghanem:** Writing – review & editing.

## Declaration of competing interest

The authors declare that they have no known competing financial interests or personal relationships that could have appeared to influence the work reported in this paper.
