# Supplementary material for: Cadmium and lead accumulation in important food crops due to wastewater irrigation: Pollution index and health risks assessment
Source: Heliyon. 2024 Jan 20;10(3):e24712. doi: 10.1016/j.heliyon.2024.e24712 (PMC10838743; doi:10.1016/j.heliyon.2024.e24712)
Supplement: Multimedia component 1 [file mmc1.docx]

**Supplementary Table 1**

Physiochemical properties of soil sampled from Faisalabad and Multan.

| **Parameters** | **Units** | **Value** | |
| --- | --- | --- | --- |
|  |  | **Faisalabad** | **Multan** |
| Sand | % | 49.50 | 54.75 |
| Silt | % | 26.00 | 30.91 |
| Clay | % | 24.50 | 14.34 |
| Texture | --- | Sandy Clay Loam (Dominant texture) | Sandy Loam (Dominant texture) |
| Saturation percentage | % |  |  |
| pH | --- | 8.34-8.66 | 8.45-8.73 |
| Electrical conductivity | dS m^-1^ | 4.89-7.23 | 4.13-7.55 |
| Carbonates | me L^-1^ | 0.5-1.35 | 0.6-1.55 |
| Bicarbonates | me L^-1^ | 4.78-8.56 | 5.22-9.34 |
| Chlorides | me L^-1^ | 7.45-15.34 | 8.01-17.66 |
| Sodium | me L^-1^ | 10.45-14.66 | 11.21-16.34 |
| Calcium + Magnesium | me L^-1^ | 9.89-13.44 | 8.99-14.09 |
| Cation exchange capacity | cmol_c_ kg^-1^ | 5.4-6.3 | 5.5-6.1 |
